# Alternative promoters control UGT2B17-dependent androgen catabolism in prostate cancer and its influence on progression

**DOI:** 10.1038/s41416-020-0749-2

**Published:** 2020-02-12

**Authors:** Eric Lévesque, Adrien Labriet, Hélène Hovington, Éric P. Allain, Luciana Melo-Garcia, Michèle Rouleau, Hervé Brisson, Véronique Turcotte, Patrick Caron, Lyne Villeneuve, Mickaël Leclercq, Arnaud Droit, Etienne Audet-Walsh, David Simonyan, Yves Fradet, Louis Lacombe, Chantal Guillemette

**Affiliations:** 1 Centre Hospitalier Universitaire de Québec (CHU de Québec) Research Center—Université Laval and Faculty of Medicine, Québec, Canada; 2 Pharmacogenomics laboratory, Centre Hospitalier Universitaire de Québec (CHU de Québec) Research Center—Faculty of Pharmacy, Laval University, Québec, Canada; 3 Statistical and Clinical Research Platform, CHU de Québec Research Center—Université Laval, Québec, Canada

**Keywords:** Prognostic markers, Prostate cancer, Epidemiology, Tumour biomarkers

## Abstract

**Background:**

Perturbation of the major UGT2B17-dependent androgen catabolism pathway has the potential to affect prostate cancer (PCa) progression. The objective was to evaluate UGT2B17 protein expression in primary tumours in relation to hormone levels, disease characteristics and cancer evolution.

**Methods:**

We conducted an analysis of a high-density prostate tumour tissue microarray consisting of 239 localised PCa cases treated by radical prostatectomy (RP). Cox proportional hazard ratio analysis was used to evaluate biochemical recurrence (BCR), and a linear regression model evaluated variations in circulating hormone levels measured by mass spectrometry. The transcriptome of *UGT2B17* in PCa was established by using RNA-sequencing data.

**Results:**

UGT2B17 expression in primary tumours was associated with node-positive disease at RP and linked to circulating levels of 3α-diol-17 glucuronide, a major circulating DHT metabolite produced by the UGT2B17 pathway. UGT2B17 was an independent prognostic factor linked to BCR after RP, and its overexpression was associated with development of metastasis. Finally, we demonstrated that distinctive alternative promoters dictate UGT2B17-dependent androgen catabolism in localised and metastatic PCa.

**Conclusions:**

The androgen-inactivating gene *UGT2B17* is controlled by overlooked regulatory regions in PCa. UGT2B17 expression in primary tumours influences the steroidome, and is associated with relevant clinical outcomes, such as BCR and metastasis.

## Background

Prostate cancer (PCa) is the most commonly diagnosed cancer in North American men.^1^ The prostate is an androgen-dependent organ, and steroid hormones contribute to PCa cell proliferation and overall survival via activation of the androgen receptor (AR).^2^ Even when a PCa patient is castrated, which leads to declined circulating testosterone levels, AR amplification, AR splice variants and proficient intratumoural steroid biotransformation pathways compensate and sustain progression.^3–5^ Indeed, survival improves upon administration of AR axis-targeting agents in castration-resistant metastatic disease,^6–9^ supporting intracrine biotransformation processes. Uridine diphospho-glucuronosyltransferase enzymes (UGTs) are responsible for the inactivation of various endogenous signalling molecules, including androgens and oestrogens.^10^ UGT enzymes are located in the membranes of the endoplasmic reticulum and glycosylate their steroid substrates with glucuronic acid,^11^ an enzymatic reaction referred to as glucuronidation. This results in glucuronide (–G) derivatives that are more hydrophilic than the parent molecules, and can be more easily excreted into bile or urine.^10^ Glucuronidation is an essential pathway for steroid inactivation and elimination in cancer cells, and is very active in the prostate.^12^ Thus, the UGT pathway participates in the regulation of the local exposure of prostate cells to steroid hormones, and this pathway has been shown to influence the risk^13,14^ and progression of PCa.^15–17^

**Fig. 1:**
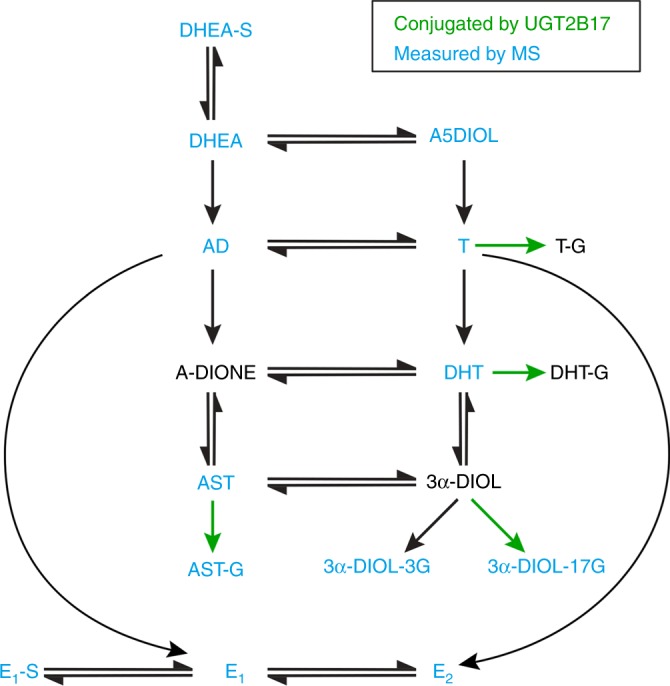
Simplified schematic representation of steroids inactivated by UGT2B17 and steroids measured in plasma of 239 PCa patients. UGT2B17 is involved in the inactivation of several C_19_ steroids as indicated. Thirteen steroids (blue labelling) were profiled by multiplex MS assays in preoperative plasma samples. DHEA-S dehydroepiandrosterone-sulfate, DHEA dehydroepiandrosterone, A5diol androstenediol, T testosterone, T-glucuronide T-G, DHT dihydrotestosterone, DHT-glucuronide DHT-G, A-dione androstanedione, AST androsterone, 4-Dione/AD androstenedione, AST-G androsterone-glucuronide, 3α-diol-3-G androstane-3α,17β-diol-3-glucuronide, 3α-diol-17G androstane-3α,17β-diol-17-glucuronide, E_1_-S oestrone-sulfate, E_1_ oestrone, E_2_ oestradiol.

UGT2B17, along with UGT2B15 and UGT2B28, is a major enzyme involved in steroid inactivation in both normal and prostatic cancer cells (Fig. 1).^18–20^ UGT2B17 shares 94% amino acid sequence identity with UGT2B15^18^ and 76% with UGT2B28.^20^ It was previously documented that *UGT2B17* mRNA expression is associated with PCa disease stages, being overexpressed in castration-resistant metastasis.^21^ More recently, the complexity of the *UGT2B17* transcriptome was exposed with at least ten alternative mRNA isoforms,^22^ but it remains unclear which transcripts are linked to disease stages.

Thus far, only one study evaluated the impact of UGT2B17 protein expression in primary tumour samples.^23^ In this study, the expression of UGT2B17 was associated with a trend towards an increased risk of biochemical failure (BCR) after radical prostatectomy (RP), and was performed using a commercially available polyclonal antibody with an undetermined specificity.^23^ Based on the significant role of UGT2B17 on steroid catabolism,^18^ and the very high protein sequence identity amongst UGT2B family members active towards steroids, our group recently developed and characterised a specific monoclonal UGT2B17 antibody^20,24,25^ to appraise the importance of this androgen catabolic pathway in PCa. We assessed (1) the relationship between UGT2B17 protein expression and clinico-pathological factors in a well-characterised cohort of 239 patients with localised disease, (2) evaluated whether UGT2B17 expression is associated with changes in the circulating steroid metabolome and (3) appraised whether UGT2B17 is an independent marker associated with the risk of PCa progression following primary curative treatment by RP. (4) We established the *UGT2B17* transcriptome in the prostate and tumours to corroborate our observations at the protein level, and (5) identified alternative promoters as a mechanism governing UGT2B17 expression in the prostate, localised PCa and metastatic disease.

## Methods

### Patients and clinical data

The study included 239 patients with localised PCa who were recruited from February 1999 to December 2002 at the L’Hôtel-Dieu de Québec Hospital, Québec, Canada.^16^ All patients had localised PCa at the time of diagnosis, and had undergone radical prostatectomy (RP) by two uro-oncologic surgeons (L.L. and Y.F.). None of the 239 patients received neoadjuvant hormone treatments. Serial prostate-specific antigen (PSA) measurements and clinical data were gathered from patient medical records during follow-up. Tissue microarrays (TMA) were prepared from the paraffin-embedded tumour samples available for all 239 patients, as described.^16^ All participants provided written informed consent, and the CHU de Quebec research ethics committee approved the research protocol (#2012-362). Table 1 lists the characteristics and descriptive statistics of the study cohort. At a median follow-up time of 182 months after prostatectomy, 73 patients experienced BCR, 15 developed metastatic disease, 10 castration resistance and 7 died from PCa.

**Table 1:** Clinical and pathological characteristics of the cohort studied.

Patients characteristics	Localised PCa (*n* = 239)
Age at diagnosis, years	
Mean	62.9
SD	6.6
Range	43.3–78.4
Follow-up median, months	182
Biochemical recurrence, *n* (%)	73 (31)
Development of metastasis	15 (6)
PSA level at diagnosis, *n* (%)	
≤ 10 ng/mL	159 (67)
> 10–20 ng/mL	62 (26)
> 20 ng/mL	17 (7)
Pathologic Gleason score, *n* (%)	
≤ 6	68 (28)
7	124 (52)
≥ 8	47 (20)
Pathologic T stage, *n* (%)	
≤ T2c	141 (59)
T3a	63 (26)
≥ T3b	35 (15)
Nodal invasion, *n* (%)	
N0	232 (97)
N+	7 (3)
Margin status, *n* (%)	
Negative	160 (68)
Positive	77 (32)

### Immunohistochemistry

Analysis of UGT2B17 expression was carried out by immunohistochemistry (IHC) staining of 5-μm-thick sections from the paraffin-embedded tumour samples in the TMA using the novel EL-2B17mAb monoclonal antibody (dilution 1:5000) and the androgen receptor (AR) antibody (sc-816; dilution 1:400; Santa Cruz, TX, USA). Two experienced lab members, H. Brisson and H. Hovington, independently scored TMA staining. They were blind with respect to tumour characteristics and clinical outcomes. The intensity of staining was scored as 0 or 1 when it was absent or negligible, respectively, and as 2 + or 3 + when the intensity was moderate or strong, respectively. Only cells with 2 + /3 + staining were considered positive. Data are presented as the mean ± standard error of the mean of the percentage of UGT2B17 and AR-positive cells.

### Plasma steroid levels

Plasma samples were collected on the morning of the surgery. Plasma steroid levels were measured by liquid chromatography–tandem mass spectrometry and gas chromatography–mass spectrometry as described.^16,26^ The measured steroids and their limits of quantification were as follows: dehydroepiandrosterone (DHEA, 0.2 ng/ml); androstenediol (A5Diol, 0.05 ng/ml); testosterone (T, 0.05 ng/ml); dihydrotestosterone (DHT, 0.01 pg/ml); androsterone (AST, 0.05 pg/ml); oestrone (E_1_, 0.005 ng/ml); oestradiol (E_2_, 0.001 ng/ml); androstenedione (AD, 0.05 ng/ml); AST-G (1 ng/ml); androstane-3α, 17β-diol-3-G (3α-diol-3-G, 0.25 ng/ml); 3α-diol-17-G (3α-diol-17G, 0.25 ng/ml); DHEA-sulfate (DHEA-S, 0.075 mg/ml); oestrone-sulfate (E_1_-S, 0.075 ng/ml). Reference steroids were purchased from Steraloids (Newport, RI, USA). Three low and three high hormone concentration quality control replicates were included in each run, and all metabolite coefficients of variation were <10%.

### Western blot analyses

Specificity of commercially available antibodies directed against UGT2B17 Abcam #ab92610 and UGT2B28 Abcam #ab156131, previously used to report UGT2B17 and UGT2B28 expression in primary PCa tumours,^23^ were tested on commercially available supersomes and UGT2B microsomal protein preparations isolated from UGT-negative HEK293 cells overexpressing each of the seven individual human UGT2B enzymes. Supersomes (0.5–1.0 µg) and microsomes (2–20 µg) were mixed with Laemmli sample buffer (Bio-Rad Laboratories, CA, USA), heated at 100 °C for 5 min, separated on a 10% SDS-PAGE gel and transferred to a nitrocellulose membrane. The membranes were blocked with PBS containing 0.2% Igepal (Sigma Aldrich, MO, USA) and 5% dry milk, probed with the UGT2B17 Abcam #ab92610 (1:500) and UGT2B28 Abcam #ab156131 antibodies (1:500) overnight and then revealed using standard protocols. The human monoclonal EL-2B17mAb towards UGT2B17 was recently described.^24^ Briefly, the EL-2B17mAb was produced by Genscript (Piscataway Rownshi, NJ, USA) using their custom monoclonal antibody production protocol.^24^ The immunogenic peptide comprised the UGT2B17 amino acids 83–99 in the substrate-binding region of the enzyme, and was selected based on specificity relative to the sequence of the other six human UGT2B enzymes.^24^

### Analysis of UGT mRNA expression

Expression of *UGT2B* transcripts was assessed in RNA-sequencing data sets of (i) normal liver (*n* = 50) and prostate (*n* = 50) tissues from The Genotype-Tissue Expression (GTEx) project (Analysis Release V7) obtained through the GTEx portal (https://www.gtexportal.org/home/) on June 18th, 2019. (ii) PCa tissues (*n* = 57) obtained from The Cancer Genome Atlas (TCGA, https://cancergenome.nih.gov/) accessed through the Genomic Data Commons Data Portal (https://portal.gdc.cancer.gov/) on June 18th, 2019. (iii) Metastatic PCa tissues (*n* = 12; GSE118435)^27^ as well as (iv) the LNCaP cell line using our GSE128749 data set. FASTQ sequence data files were realigned to the complete human *UGT*-annotated loci that include all novel alternatively spliced UGT variants, using kallisto v0.44.0.^22,28^ Correlations between *UGT2B17* mRNA expression in primary tumours vs metastasis were performed in three additional public data sets.^29–31^ For transcript validation, the total RNA from LNCaP cells was reverse-transcribed using SuperScript IV Reverse Transcriptase (Invitrogen, MA, USA) as recommended. For the first PCR, 50 ng of cDNA served as a template in standard PCR reactions. PCR products were used at a final 1:50 dilution in nested PCR reactions. Each round of PCR comprised 40 cycles and conducted with AmpliTaq Gold DNA polymerase (Applied Biosystems, MA, USA). Elongation was 20 s for the first round and 15 s for the nested PCR. Primers and annealing temperatures are shown in Supplementary Table 1. Direct Sanger sequencing verified the identity of PCR products.

### Genetic analysis

Blood samples were collected before patients underwent RP. DNA was extracted from peripheral mononuclear cells and purified using QIAamp DNA Blood Mini kit reagents (QIAGEN, Ontario, Canada). The *FOXA1* marker rs59678213, influencing UGT2B17 protein expression in the liver and cancer cell lines,^24,32^ was genotyped by PCR followed by sequencing using Sequenom iPLEX matrix-assisted laser desorption/ionisation–time-of-flight mass spectrometry (Sequenom, CA, USA) as described.^26^

### Luciferase reporter gene assays

The pGL3 construct containing the *UGT2B17_v1* promoter was obtained as described.^33^ The shorter *UGT2B17_n3* promoter constructs comprising nucleotides (nt) –2422 to –1290 (coordinates relative to the translation initiation codon ATG) were generated from the pGL3-*UGT2B17_v1* promoter plasmid by site-directed mutagenesis using the Q5 Site-Directed Mutagenesis Kit (New England Biolabs, Ontario, Canada) as recommended. A longer *UGT2B17_n3* promoter region (nt –4104 to –1290) was PCR-amplified with *Phusion* DNA polymerase from LNCaP genomic DNA, digested with *Mlu*I and *Xho*I restriction enzymes and inserted into pGL3 basic vector (Promega, WI, USA) digested with the same enzymes, using the Rapid DNA Ligation Kit (Roche, Quebec, Canada). The *UGT2B17_n2* promoter region (nt –10301 to –7466) was similarly amplified and cloned. All primer sequences and annealing temperatures are provided in Supplementary Table 1. Sanger sequencing verified all constructs. CMV-renilla plasmid (Promega, WI, USA) was used as an internal control for luciferase reporter gene assays, which were conducted in LNCaP, LAPC4 and 22Rv1 PCa cell lines, as reported.^24^ Cells were grown as described, except that they were serum-starved for 3 days prior to transfection using 2% SVFA in place of foetal bovine serum. Treatment with either R1881 (1 nM, steraloids) or vehicle (ethanol) was initiated at the time of transfection in serum-deprived media for 2 days, after which luciferase expression was assessed as described.^24^

### Statistical analysis

Clinical and pathological factors comprising Gleason scores at diagnosis, margin and nodal status were included and considered as categorical variables. Only PSA at diagnosis was considered as a continuous variable and expressed as a mean ( ± standard error), 95% confidence interval or median (first and third quartile). Correlation between variables was assessed using Spearman correlation. The relationship between TMA staining and clinical or pathological factors was tested using Mann–Whitney *U* test or Kruskal–Wallis test followed by Dunn’s post hoc tests on each pair of groups. Fisher’s tests were used to assess the association between UGT2B17 expression and the categorical variables, and were followed by logistic regression analysis. Hormone levels were assessed as means, and their variations were expressed as 95% confidence interval and/or the standard deviation of the mean. To assess the association between UGT2B17 expression (<25% vs ≥ 25%) and hormone levels, blood steroid concentrations were subjected to multivariable-generalised linear regression analysis adjusted for age and smoking status, as performed in studies similar in scope.^34–36^ Adjusted hazard ratios were obtained using multivariable Cox proportional hazard regression modelling, including age, PSA (continuous) and Gleason scores at diagnosis, pathological T staging (extracapsular extension (pT3a) and seminal vesicle invasion (pT3b)), margin status, nodal status and smoking information. Two-sided *P*-values were used and considered statistically significant at *P* ≤ 0.05. All analyses were done using SAS 9.4, R v3.5.1 or GraphPad Prism 5.

## Results

### UGT2B17 subcellular localisation is altered in PCa compared with normal peritumoural glands

**Fig. 2:**
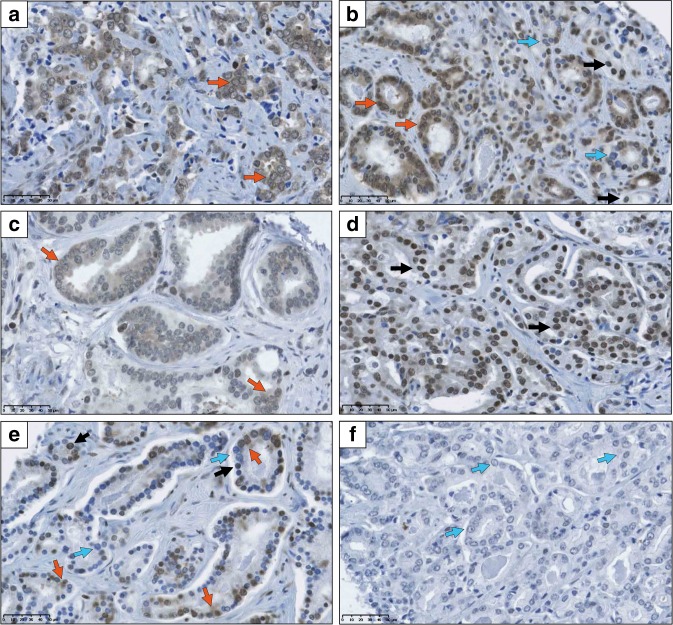
UGT2B17 localisation in prostate tumours. Immunohistochemistry staining using the EL-2B17mAb was performed on primary tumours (**a**–**f**). UGT2B17 expression in tumours is observed in both cytoplasmic and nuclear/perinuclear compartments. Cytoplasmic expression of UGT2B17 is ~90–100% in (**a**), ~75% in (**b**), ~20–30% in (**c**) and ~5–10% in (**d**, **e**). Nuclear expression of UGT2B17 is ~90% in (**d**) and ~30% in (**e**). A tumour lacking UGT2B17 cytoplasmic and nuclear staining is depicted in (**f**). Basal and stromal cells were devoid of staining. Black arrows show positive nuclear expression, orange arrows show nuclear and cytoplasmic expression. In **e** and **f**, blue arrows show negative nuclear and cytoplasmic UGT2B17 expression.

We initially investigated the expression and cellular distribution of UGT2B17 in benign prostatic tissue and in prostate tumours using the monoclonal anti-UGT2B17 antibody (EL-2B17mAb), for which the specificity was recently reported.^24^ In normal prostate epithelial cells (*n* = 3), UGT2B17 displayed mostly a nuclear/perinuclear staining (Supplementary Table 2). In contrast, UGT2B17 staining was detected in both the nucleus and the cytoplasm of neoplastic epithelial cells (Fig. 2a–e). No UGT2B17 staining of basal or stromal cells was observed in prostate tissues. An example of UGT2B17-negative staining is provided in Fig. 2f.

### UGT2B17 expression in primary tumour and its relation to prognostic factors and clinical outcomes

**Fig. 3:**
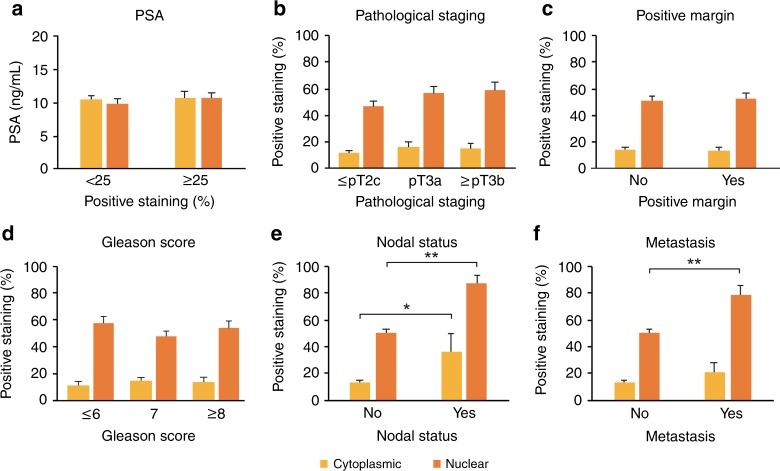
UGT2B17 expression in primary tumours and PCa prognostic factors (*n* = 239). Association of UGT2B17 cytoplasmic and nuclear expression with (**a**) PSA level, (**b**) pathological staging, (**c**) the presence of positive margin, (**d**) Gleason scores, (**e**) nodal status and (**f**) metastasis. PSA prostate-specific antigen. Data are presented as mean ± standard error of the mean. Comparisons between groups were performed using a two-tailed Mann–Whitney *U* test or a Kruskal–Wallis test followed by Dunn’s post hoc test. *P < 0.05; **P < 0.01.

With a median follow-up time of 182 months, BCR and metastatic disease occurred in 73 and 15 patients, respectively. The distribution of UGT2B17 nuclear and cytoplasmic staining in the overall cohort is shown in Supplementary Fig. 1. In primary tumours, a weak correlation was observed between UGT2B17 nuclear and cytoplasmic staining (*r*_s_ = 0.14; *P* = 0.025). UGT2B17 expression was not associated with PSA levels at diagnosis, pathological Gleason scores, pathological T staging and margin status (Fig. 3a–d). Conversely, both UGT2B17 cytoplasmic and nuclear staining in primary tumours were associated with node-positive disease at surgery, and nuclear staining with the development of metastasis during follow-up (Fig. 3e, f). UGT2B17 cytoplasmic staining showed a non-significant trend towards an association with development of metastasis. Another representation of UGT2B17 nuclear boxplot distribution associated with nodal status and development of metastatic disease is shown in Supplementary Fig. 2. In multivariable proportional hazard Cox models, UGT2B17 cytoplasmic staining ≥ 25% was associated with BCR with a HR of 1.85 (95% CI: 1.05–3.25; *P* = 0.033), whereas UGT2B17 nuclear staining ≥ 25% did not reach significance (HR = 1.38, 95% CI: 0.80–2.34; *P* *=* 0.246). Using different cut-off values, cytoplasmic staining of ≥25–74% is associated with a HR of 2.04 (95% CI: 1.14–3.66; *P* = 0.017). Only 12 patients had a cytoplasmic staining ≥75%, and therefore limit the interpretation of this higher cut-off value (HR = 1.01, 95% CI: 0.24–4.29; *P* = 0.985). A nuclear staining of ≥25–74% is associated with a HR of 1.11 (95% CI: 0.57–2.16; *P* = 0.766) and ≥ 75% with a HR of 1.60 (95% CI: 0.89–2.88; *P* = 0.116). In agreement with our findings observed at the protein level, *UGT2B17* mRNA expression was also increased in metastasis compared with primary tumours in three independent data sets (Supplementary Fig. 3).^29–31^ A multivariable analysis of the Taylor data set (*n* = 131)^30^ further suggested that primary PCa patients with elevated *UGT2B17* mRNA level had superior BCR rates (HR = 2.4; 95% CI: 1.00–5.67; *P* = 0.051).

### UGT2B17 expression in relation to AR expression and the functional variation in the proximal promoter *FOXA1*-binding site

Based on previous findings of UGT2B17 regulation involving the AR in the PCa cell line LNCaP,^37,38^ we examined the potential correlation between AR and UGT2B17 expression in clinical samples. Western blot analysis demonstrated the specificity of the AR (sc-816) antibody used in this study (Supplementary Fig. 4). Examples of immunohistochemistry staining performed on tumours using the AR antibody are shown in Supplementary Fig. 5. In primary tumours, weak but positive correlations were observed between the AR and cytoplasmic (*r*_s_ = 0.33; *P* < 0.0001; *r*^2 ^= 12.4%) and nuclear (*r*_s_ = 0.20; *P* = 0.005; *r*^2^ = 3.76%) UGT2B17 staining (Supplementary Fig. 6). When using an AR cut-off value of 25%, the mean UGT2B17 nuclear expression is 38% (95% CI: 29–46) vs 57% (95% CI: 50–64) in the AR < 25% vs AR ≥ 25% tumours, respectively (*P* = 0.0003). The mean UGT2B17 cytoplasmic expression is 6% (95% CI: 3–9) vs 19% (95% CI: 14–24) in the AR < 25% vs AR ≥ 25% tumours, respectively (*P* < 0.0019). Associations between the AR and clinical and pathological factors are shown in Supplementary Fig. 7. We also observed no significant association between the functional germline *FOXA1* rs59678213 variant shown to impact *UGT2B17* hepatic expression^24^ and in LNCaP cells^24,32^ (Supplementary Fig. 8).

### UGT2B17 expression in primary PCa in relation to the circulating steroidome

Mean hormone levels of 13 steroid hormones measured in the circulation of PCa patients are provided in Supplementary Table 3. We observed that high UGT2B17 cytoplasmic staining (≥ 25% vs <25%) was associated with superior levels of DHEA-S by 16% (0.92 vs 1.07 µg/mL; *P* = 0.042), 28% for E_1_-S (0.50 vs 0.64 ng/mL; *P* = 0.033) and 21% higher levels of the major DHT metabolite 3α-diol-17G (3.74 vs 4.52 ng/mL; *P* = 0.042) (Table 2). UGT2B17 nuclear staining (≥ 25% vs < 25%) was associated with 11% lower levels of E_1_ (31.97 vs 28.59 ng/mL; *P* = 0.044). When considering both hormones and UGT2B17 staining as continuous variables, a positive correlation was observed between nuclear staining and AST-G (*r*_s_ = 0.16; *P* = 0.015,) and a trend was observed for oestradiol (*r*_s_ = –0.13; *P* = 0.061).

**Table 2:** Association between UGT2B17 expression (<25% vs ≥25%) in primary tumours and circulating steroid levels (*n* = 239).

Steroids	Staining score (%)	UGT2B17 nuclear staining				UGT2B17 cytoplasmic staining			
		*n*	Mean	95% CI	*P* value	*n*	Mean	95% CI	*P* value
DHEA-S (μg/mL)	<25	75	0.97	0.83–1.10	0.386	172	0.92	0.84–1.00	**0.042**
	≥25	148	0.95	0.86–1.04		51	1.07	0.88–1.26	
DHEA (ng/mL)	<25	75	1.99	1.65–2.32	0.286	171	1.94	1.73–2.15	0.810
	≥25	147	1.89	1.68–2.10		51	1.87	1.56–2.17	
A5diol (pg/mL)	<25	75	615.60	551.59–679.61	0.490	171	593.68	545.44–641.91	0.503
	≥25	147	593.98	540.37–647.59		51	626.79	545.85–707.73	
AD (ng/mL)	<25	69	0.75	0.65–0.85	0.823	153	0.68	0.62–0.74	0.271
	≥25	134	0.67	0.61–0.73		50	0.74	0.63–0.85	
T (ng/mL)	<25	75	4.11	3.79–4.43	0.676	171	4.05	3.81–4.28	0.968
	≥25	147	4.02	3.75–4.28		51	4.06	3.64–4.47	
DHT (pg/mL)	<25	75	346.56	311.26–381.85	0.922	171	344.47	320.34–368.60	0.741
	≥25	147	345.88	319.39–372.36		51	351.59	307.12–396.06	
AST (pg/mL)	<25	75	202.28	181.78–222.79	0.922	172	204.04	179.87–228.21	0.966
	≥25	148	204.28	176.14–232.42		51	202.15	171.66–232-64	
E_1_-S (ng/mL)	<25	75	0.59	0.50–0.68	0.208	172	0.50	0.45–0.56	**0.033**
	≥25	148	0.51	0.44–0.57		51	0.64	0.50–0.78	
E_1_ (pg/mL)	<25	75	31.97	28.83–35.11	**0.044**	172	28.97	27.24–30.70	0.073
	≥25	148	28.59	26.77–30.41		51	32.28	28.33–36.23	
E_2_ (pg/mL)	<25	75	21.35	19.51–23.18	0.821	172	21.13	17.52–24.75	0.634
	≥25	148	20.50	16.33–24.66		51	19.61	17.55–21.66	
AST-G (ng/mL)	<25	75	32.04	26.08–38.01	0.384	173	33.53	30.54–36.52	0.566
	≥25	149	35.03	31.73–38.33		51	35.72	27.45–44.00	
3αDiol-3-G (ng/mL)	<25	75	2.06	1.79–2.34	0.091	173	1.84	1.69–1.99	0.148
	≥25	149	1.81	1.64–1.98		51	2.08	1.69–2.47	
3αDiol-17G (ng/mL)	<25	75	3.96	3.35–4.56	0.820	173	3.74	3.45–4.04	**0.042**
	≥25	149	3.90	3.54–4.26		51	4.52	3.57–5.47	

All *P* values estimated by multivariable linear regression models adjusted for age and smoking status.

*P* values < 0.05 are indicated in bold.

### The *UGT2B17* transcriptome in normal prostate, primary PCa and metastasis

**Fig. 4:**
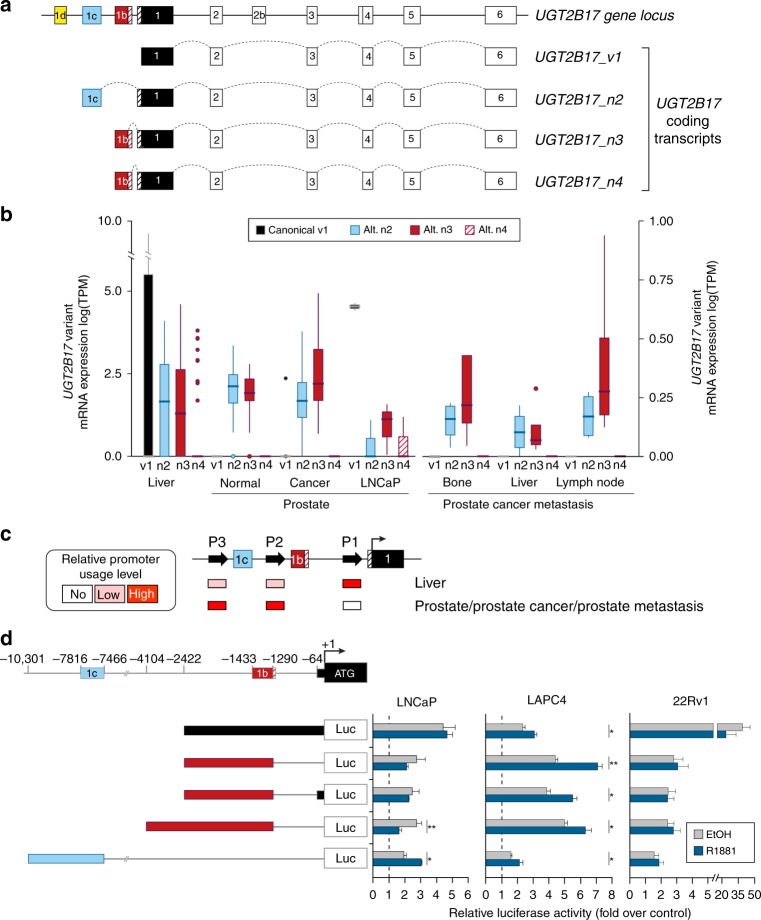
Transcriptional regulation of *UGT2B17* by alternative promoters. **a** Schematic representation of the *UGT2B17* gene and transcripts encoding the UGT2B17 enzyme. *UGT2B17_v1* represents the canonical transcript (NCBI RefSeq accession number: NM_001077^18^). *UGT2B17_n2–n4* (as described in ref. ^22^) is the main expressed alternative (alt.) transcript encoding the UGT2B17 enzyme in prostate tissues. These alt. transcripts include an additional exon 1b or exon 1c. **b** Expression of *UGT2B17* mRNA variants based on GTex and TCGA RNA-sequencing data. **c** Usage of *UGT2B17* gene alt. promoters P2 and P3 relative to the canonical promoter P1 in the liver and prostate tissues based on RNA-sequencing data. no: expression undetected. The schematic representation highlights the relative promoter usage within each sample studied. **d** Transcriptional influence of alt. promoters on *UGT2B17* expression in PCa cell lines. Luciferase expression driven by the specified promoter regions was assessed in LNCaP, LAPC4 and 22Rv1 grown with vehicle (ethanol) or R1881 (1 nM). Each experiment was conducted at least three times in triplicates. *P < 0.05; **P < 0.01.

Given the similarity between UGT2B family members and the complexity of the *UGT2B17* transcriptome, we evaluated their expression in normal prostate, primary and metastatic PCa. Similar to normal prostate tissue, prostate tumours expressed several *UGT2B* mRNAs, including *UGT2B17* but also *UGT2B4*, *UGT2B7*, *UGT2B10*, *UGT2B11*, *UGT2B15* and *UGT2B28* (Supplementary Fig. 9). Based on the mapping of RNA-sequencing reads on the previously assembled *UGT* transcriptome that includes ten alternative *UGT2B17* transcripts, we evidenced a predominant expression of the canonical isoform (*v1*) in human liver samples and in the PCa LNCaP cell model (Fig. 4a, b). By contrast, we observed a prevalent expression of alternative *UGT2B17*_*n2*, having an additional exon 1b, and *UGT2B17*_*n3* having an additional exon 1c in normal prostate, primary tumours and metastasis, with a minimal or undetectable expression of the canonical *v1* mRNA (Fig. 4a, b). Expression of *UGT2B17*_*n2*–*n4* transcripts was validated by PCR amplification and sequencing (Supplementary Fig. 10).

### Alternative promoters control *UGT2B17* expression in PCa

Whereas alternative transcripts *n2*, *n3* and *n4* all code for the full-length UGT2B17 enzyme as does the canonical transcript *v1*, they include an additional 5′ exon, suggesting a regulation of their expression by alternative promoters (P2 and P3) rather than by the proximal promoter P1 in PCa (Fig. 4). The functionality of alternative promoters driving the expression of *UGT2B17* was tested using luciferase constructs engineered to reflect combinations of alternative promoters and the associated spliced alleles. Luciferase assays conducted in commonly used PCa cell models (LNCaP, LAPC4 and 22Rv1) support the transcriptional potential of each promoter region in these cell lines (Fig. 4d), and an impact of the synthetic androgen R1881 treatment on gene expression. Transcription from promoter P1 was active in LNCaP, LAPC4 and 22Rv1 (Fig. 4d). By contrast, in LAPC4, P1 and promoter 2 (P2) drove most gene expression of luciferase constructs, whereas the most distal P3 was less active in these cells (Fig. 4d). In LNCaP cells, P2 was repressed by androgens and P3 was inducible. In LAPC4, all three promoters were induced by R1881, whereas the results did not reach the level of significance in 22Rv1 (Fig. 4d).

## Discussion

UGT2B17 is a key enzyme involved in the control of steroid metabolism in normal prostate and PCa cells.^18^ In our study, we evidenced a differential UGT2B17 protein expression and subcellular distribution in primary PCa compared with normal glands, and the association of cytoplasmic staining with positive nodes at surgery, circulating steroid levels and an independent risk factor of BCR after RP. Although the number of metastatic events were limited, UGT2B17 protein expression in primary tumours was further associated with the development of metastasis during follow-up, which represents a surrogate marker of PCa-specific survival in this patient population.^39^

Two prior studies evaluated at the protein level impact UGT protein expression in primary tumour samples, and established correlations with clinical outcomes after prostatectomy.^16,23^ The first study by Belledant et al.^16^ utilised a specific polyclonal UGT2B28 antibody, and showed that higher expression levels in primary tumours were associated with more aggressive PCa and greater risk of progression. The second study by Grant et al.^23^ used commercial UGT2B antibodies and appraised the impact of both UGT2B17 and UGT2B28 expression on clinical outcomes after prostatectomy. The authors reported a trend towards an association for BCR with UGT2B17 expression, and no association with UGT2B28.^23^ However, the commercial UGT2B17 and UGT2B28 antibodies displayed cross-reactivity with other UGT2Bs expressed in prostate tumours based on our specificity screen (Supplementary Fig. 11). Therefore, it is likely that this cross-reactivity confounds the signal attributed to the UGT2B17 or UGT2B28 proteins in this previous report,^23^ and presumably explains divergence of findings among studies. This is not unexpected considering the high sequence identity between UGT2B protein members,^24^ limiting the value of several tools to study UGT expression in clinical specimens.

Immunohistochemistry data revealed nuclear/perinuclear staining in normal prostatic epithelial cells, whereas both positive cytoplasmic and nuclear staining were observed in tumours. This is comparable to observations with UGT2B28 in similar tissues, besides the fact that UGT2B28 is also expressed in basal and stromal cells.^16^ UGT2B17 cytoplasmic staining in cancer cells was associated with the risk of progression, whereas nuclear staining did not reach the level of significance. The association of cytoplasmic UGT2B17 with higher circulating levels of DHEA-S (~15%), E_1_-S ( >20%) and 3α-diol-17G ( >20%), a major DHT metabolite found in circulation, and a main substrate of the UGT2B17 enzyme,^40^ suggests that the cytoplasmic UGT2B17 protein is likely enzymatically active in primary tumours, and may be responsible, at least in part, for these changes for 3α-diol-17G produced in the prostate and then released in circulation.^18^ Nuclear UGT2B17 staining was associated with lower E_1_ levels, suggesting a distinct impact of UGT2B17 on the hormonal profile, depending on its cellular localisation. Further studies will be necessary to address precisely the impact of the cellular localisation on UGT2B17 enzymatic function.

Alternative splicing events, potential post-translational modifications and/or interactions with specific and unidentified binding partners, may partly explain the shift in subcellular distribution of UGT2B17 from the nucleus in normal cells to the cytoplasm observed in cancer cells. The transcriptome of *UGT2B17* is complex and includes at least ten alternative mRNAs in human tissues.^22^ We revealed a distinct *UGT2B17* transcription profile in the prostate compared with human liver and LNCaP cells. Whereas alternative *UGT2B17_n2* and *n3* mRNAs were the predominant transcripts expressed in normal and PCa tissues, the canonical mRNA *UGT2B17_v1* was not detected. Both *UGT2B17_n2* and _*n3* mRNAs possess the same open-reading frame as *UGT2B17_v1*, and code for an active UGT2B17 enzyme in the prostate. Our observations also indicate that the expression of *UGT2B17_n2* and _*n3* is under the control of upstream alternative regulatory regions (P2 and P3) different from P1 involved in the expression of the canonical *UGT2B17_v1* (Fig. 4c). In agreement with alternative promoters controlling UGT2B17 in PCa, no correlation between the proximal *FOXA1* polymorphism located in P1 of the *UGT2B17* gene and protein levels was observed in prostate tissues, in contrast to LNCaP cells.^24,32^ This is consistent with the predominant expression of canonical *UGT2B17_v1* in LNCaP cells. The precise underlying regulatory mechanisms involved in the prostate-specific alternative promoter and splicing programmes, which result in the inclusion of untranslated exons generating transcripts *n2* and *n3*, will need to be addressed in future studies, and are beyond the scope of this study. These observations raise the possibility that the predominance of the canonical *UGT2B17_v1* transcript, under the control of the AR axis and repressed by androgens in LNCaP cells,^37,41^ represents a sporadic subclonal evolution not representative of UGT2B17-regulatory mechanisms in PCa. Indeed, given that localised and metastatic tissues express alternative *UGT2B17* mRNAs and no *v1*, combined with rising levels of UGT2B17 with increasing AR levels in primary tumours, it raises concerns about whether LNCaP constitutes an appropriate model to further study the *UGT2B17_n2* and *_n3* regulation observed in PCa.

A proteomic study further supports the concept that increased UGT2B expression is associated with PCa aggressiveness.^17^ Previous gene expression studies have also established that *UGT2B17* mRNA expression is upregulated in metastasis,^21,42,43^ implying that *UGT2B17* expression is enhanced in aggressive cancer cells. Recently, biological explanations underlying these associations between UGT and disease phenotype have begun to emerge. UGT2B17 was shown to stimulate cancer cell proliferation, invasion and progression to castration-resistant PCa in a mouse xenograft model after prolonged androgen deprivation.^44^ In this study, the action of UGT2B17 was mediated by the activation of the c-Src kinase, enhancing ligand-independent AR signalling.^44^ However, in the latter study, the correlation between UGT2B17 tumour expression and clinical outcomes was not evaluated in men with PCa. Still, the absence of a link with PSA levels and pathological Gleason scores raised the possibility that UGT2B17 may also have uncharacterised roles in cancer progression beyond androgen signalling.^45^ This possibility is supported by the observation that increased UGT2B17 expression was also associated with more aggressive chronic lymphocytic leukaemia.^46,47^ Further studies are thus required to evaluate whether the impact of UGT2B17 on disease progression extends beyond the AR axis in PCa.^44^

The strengths of our study include the utilisation of a monoclonal UGT2B17 antibody with no cross-reactivity with other UGT enzymes, a well-characterised PCa patient cohort with detailed clinical and pathological data available from prostatectomy specimens, significant follow-up time (>15 years) to assess clinical outcomes in localised disease, measurement of steroid hormones by gold-standard mass spectrometry and supporting data from independent data sets. However, our study has the following limitations: (1) the associations between UGT expression in tumours and intratumoural androgen levels were not performed, (2) the limited number of events for clinical endpoints such as nodal status and metastasis and (3) the insufficient sample size to analyse simultaneously the impact of both UGT2B17 and UGT2B28 on disease characteristics and progression. In-depth characterisation of the new regulatory mechanisms governing UGT2B17 expression in PCa is also beyond the scope of this study.

In conclusion, UGT2B17 is a crucial enzyme involved in steroid catabolism in PCa, and its tumour expression level appears to influence the outcomes of PCa patients. Based on our findings, the relationship between UGT2B17 and cancer progression appears even more complex than originally thought, with a differential localisation of the protein in tumour cells compared with normal glands, and a complex transcriptional regulation dependent on alternative promoters and extensive alternative splicing events. Given our observations^16^ and those of others,^17,21,44^ it is highly plausible that UGT2B17 overexpression delineates diseases more likely to disseminate. Additional studies are required to further evaluate whether the role of UGT2B17 in PCa extends beyond androgen catabolism and signalling, and to decipher the regulatory mechanisms prevailing in normal prostate, primary PCa tumours and throughout metastatic dissemination.

## Supplementary information

Supplementary Tables 1-3.

Supplementary Figures 1-11.

## Data Availability

The data sets analysed during this study are available from the corresponding author on reasonable request.
