# Supplementary material for: Alternative promoters control UGT2B17-dependent androgen catabolism in prostate cancer and its influence on progression
Source: Br J Cancer. 2020 Feb 12;122(7):1068–76. doi: 10.1038/s41416-020-0749-2 (PMC7109100; doi:10.1038/s41416-020-0749-2)
Supplement: Supplementary file 1 — Supplementary Tables 1-3. [file 41416_2020_749_MOESM1_ESM.docx]

**Supplementary Table 1**. **Primers used in this study.**

| Description | Primer (5’ 🡪 3’) | Annealing |
| --- | --- | --- |
| *UGT2B17_n3* -2242+5’UTR-F | ATATAAGTATGAGAAATGACAG | 56°C |
| *UGT2B17_n3* -2242+5’UTR-R | TCTTTACAGAGAACTATAAAAC |  |
| *UGT2B17_n3* -2242-F | AGGGCTCGAGATCTGCGA | 66°C |
| *UGT2B17_n3* -2242-R | TCTTTACAGAGAACTATAAAACCACTGTC |  |
| *UGT2B17_n2* -10301-F | CTAGCAGACGCGTGAGATCCTAGTAGGAGGTTTTGGC | 63°C |
| *UGT2B17_n2* -10301-R | CTAGCAGCTCGAGCAAGTTCCAGATGTCCAGACTC |  |
| *UGT2B17_n3* -4104-F | CTAGCAGACGCGTCACGCCCTCATAATCGTCTT | 57°C |
| *UGT2B17_n3* -4104-R | CTAGCAGCTCGAGTCTTTACAGAGAACTATAAAACC |  |
| 1^st^ PCR *UGT2B17_n2*-F | CGCTCCTGGGTAGGAAATTG | 55°C |
| 1^st^ PCR *UGT2B17_n2*-R | ACACCAGCACCTTTCCACAA |  |
| Nested PCR *UGT2B17_n2*-F | TGGAACACCTCACCAGATCA | 55°C |
| Nested PCR *UGT2B17_n2*-R | ACTGAGCTGCATCAGCAGAA |  |
| 1^st^ PCR *UGT2B17_n3/n4*-F | TTTAATGAGTGCCTGGGTGC | 60°C |
| 1^st^ PCR *UGT2B17_n3/n4*-R | ACACCAGCACCTTTCCACAA |  |
| Nested PCR *UGT2B17_n3/n4*-F | GCTGAGGCCTAAAATGGCATC | 60°C |
| Nested PCR *UGT2B17_n3/n4*-R | ACTGAGCTGCATCAGCAGAA |  |

**Supplementary Table 2. UGT2B17 nuclear and cytoplasmic staining in three normal prostate samples.**

| **Prostate Samples** | **Nuclear Staining** | **Cytoplasmic staining** |
| --- | --- | --- |
| PN-1131  PN-1139  PN-1142 | 75%  75%  90% | 0%  0%  10% |

**Supplementary Table 3. Concentrations of circulating steroids observed in PCa cases (n=239) at time of surgery.**

| **Hormones (units)** | **Mean (standard error)** | **95% CI** |
| --- | --- | --- |
| DHEA-S (μg/mL)  DHEA (ng/mL)  A5diol (pg/mL)  AD (ng/mL)  DHT (pg/mL)  Testo (ng/mL)  AST (pg/mL)  E_1_-S (ng/mL)  E_1_ (pg/mL)  E_2_ (pg/mL)  3α-Diol-17G (ng/mL)  3α-Diol-3G (ng/mL)  AST-G (ng/mL) | 0.95 (0.04)  1.92 (0.09)  603.10 (20.82)  0.70 (0.03)  346.94 (10.62)  4.06 (0.10)  203.47 (9.96)  0.54 (0.03)  29.65 (0.81)  20.78 (1.42)  3.91 (0.16)  1.89 (0.07)  33.99 (1.48) | 0.88–1.03  1.74-2.09  562.08-644.12  0.64-0.75  326.02-367.86  3.86-4.26  183.84-223.10  0.49-0.59  28.05-31.24  17.99-23.58  3.60-4.22  1.75–2.03  31.07-36.92 |

CI: confidence interval.
