# Supplementary material for: Alternative promoters control UGT2B17-dependent androgen catabolism in prostate cancer and its influence on progression
Source: Br J Cancer. 2020 Feb 12;122(7):1068–76. doi: 10.1038/s41416-020-0749-2 (PMC7109100; doi:10.1038/s41416-020-0749-2)
Supplement: Supplementary file 2 — Supplementary Figures 1-11. [file 41416_2020_749_MOESM2_ESM.pdf]

Supplementary Figure 1. Boxplot distribution of UGT2B17 cytoplasmic and nuclear staining in the studied PCa cohort (n=239). Rectangles represent 95% confidence intervals, diamonds indicate the mean, horizontal bars show the median, circles designate individuals outside the 95% confidence interval and error bars denote standard error of the mean.

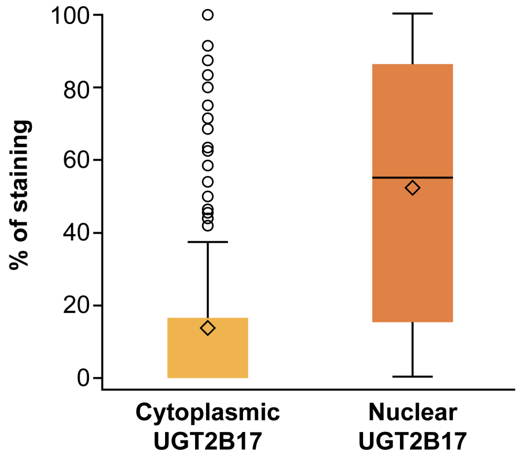

Supplementary Figure 2. Boxplot distribution of UGT2B17 nuclear staining and its association with A) Nodal status ($P < 0.01$) and B) Metastasis ($P < 0.01$). Rectangles represent 95% confidence intervals, diamonds indicate the mean, horizontal bars show the median, and error bars denote standard error of the mean.

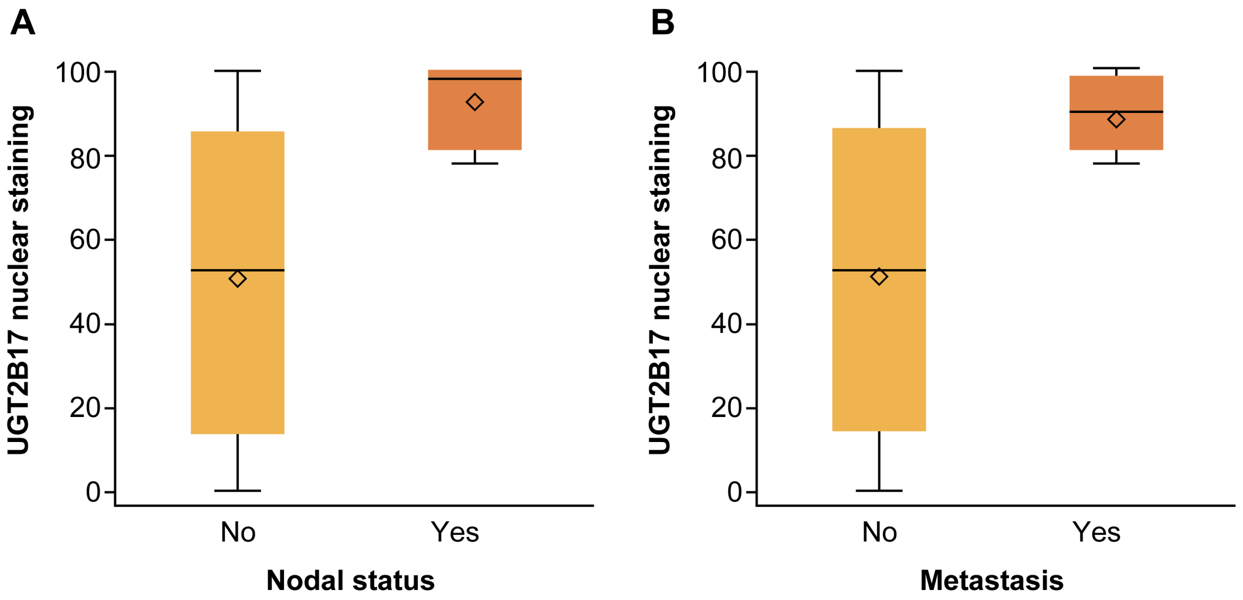

Supplementary Figure 3. *UGT2B17* mRNA is overexpressed in metastasis compared to primary tumors. Gene expression levels of *UGT2B17* were obtained from microarrays data from (A) Taylor (29); (B) Chandran (28); (C) Grasso (30). Data are presented as the mean plus or minus standard error of the mean. Comparisons between experimental groups were performed by a Mann-Whitney U test. *$P < 0.05$. FASTQ data were not available to align reads from these projects to the new UGT transcriptome preventing us to fully address the presence of alternative *UGT2B17* transcripts in metastatic diseases.

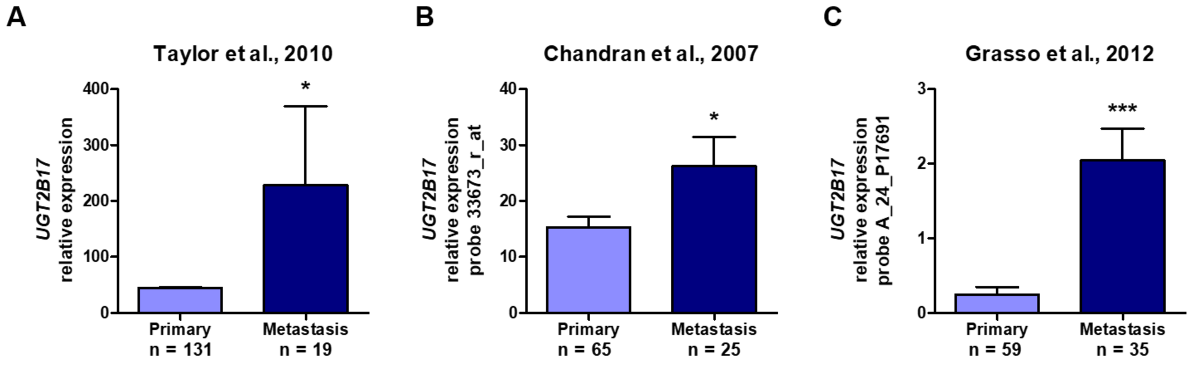

Supplementary Figure 4. Western blot analysis showing the specificity of the AR antibody (sc-816) used in this study on AR-positive PCa cells (LNCaP) and AR-negative PCa cells (PC3 and DU145). In the upper panel, 100ug of whole cell lysates of LNCaP, with and without 24h treatment with the synthetic androgen (10nM R1881), and from PC3 and DU145 cells were analyzed. Tubulin levels are shown as a loading control in the bottom panel. Arrows on the right of each panel identify molecular weights of the protein detected.

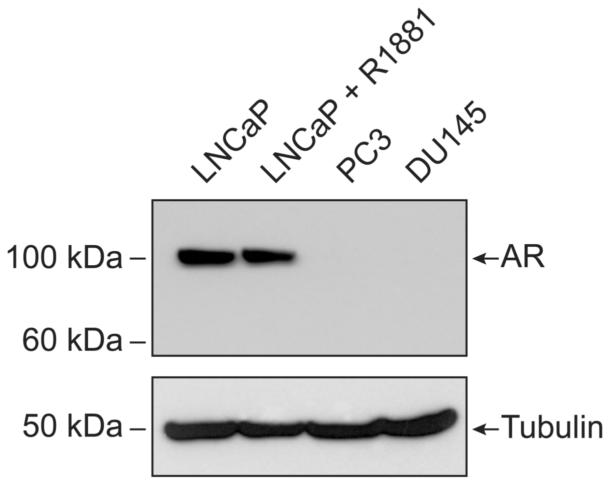

Supplementary Figure 5. Examples of immunohistochemistry staining using the AR antibody (sc-816) on normal prostate (A) and primary tumors (B-F). A) High nuclear expression of the AR (black arrows) and no expression in basal cells (blue arrows) in the normal prostate. In tumor samples, expression of the AR is ~90-100% in B), ~75% in C), ~5-10% in D), barely detectable in E) and absent in F). Black arrows = high expression; orange arrows = moderate expression; yellow arrows = low expression; blue arrows = none.

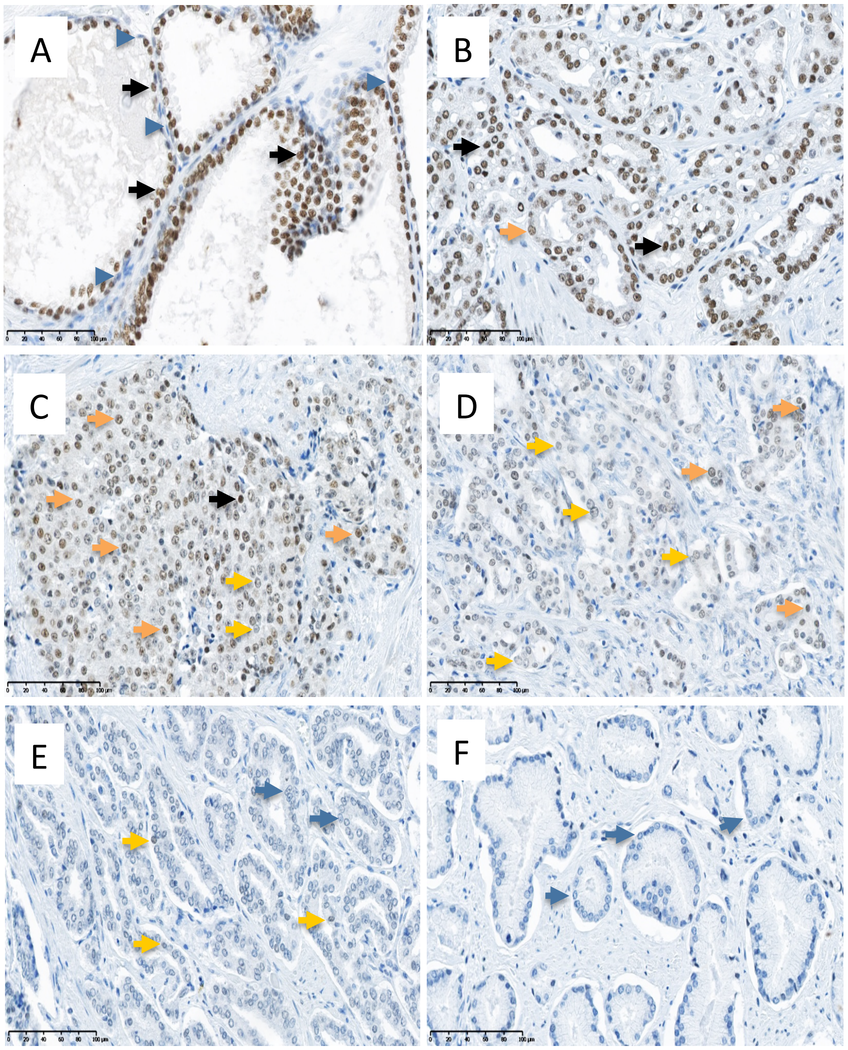

Supplementary Figure 6. Spearman correlations between UGT2B17 cytoplasmic (A) and nuclear (B) staining with the androgen receptor (AR) performed in our TMA dataset. TMA staining was performed using the EL-2B17mAb and the AR (sc-816; Santa Cruz, Texas, USA) antibodies as indicated in the Materials and Methods section.

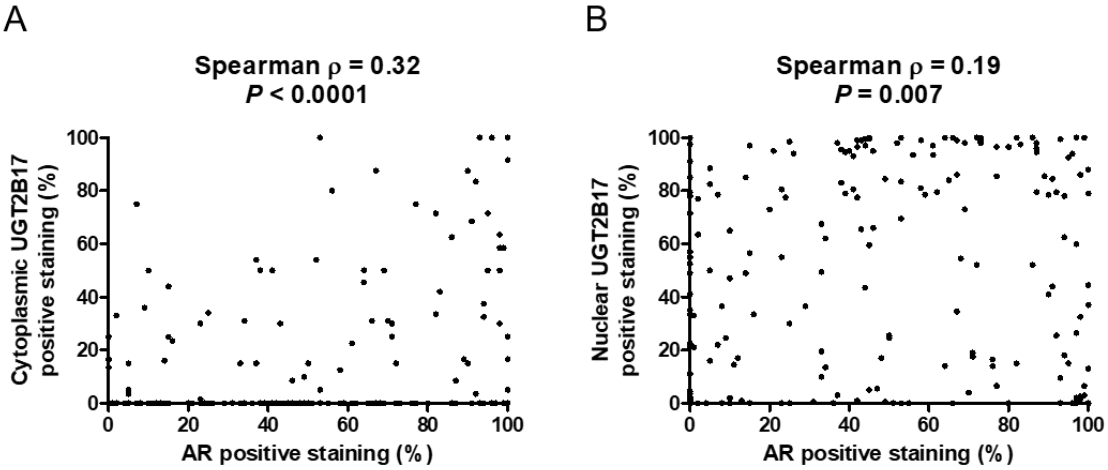

Supplementary Figure 7. AR expression in primary tumors and PCa prognostic factors. Association of AR expression with (A) PSA level, (B) pathological staging, (C) the presence of positive margin, (D) Gleason scores, (E) nodal status and (F) metastasis. TMA staining was performed using the AR antibodies as indicated in the Materials and Methods section. PSA: prostate specific antigen. Data are presented as mean ± standard error of the mean. Comparisons between groups were performed using a two-tailed Mann-Whitney U-test or a Kruskal-Wallis test followed by a Dunn's post-hoc test. **P* < 0.05.

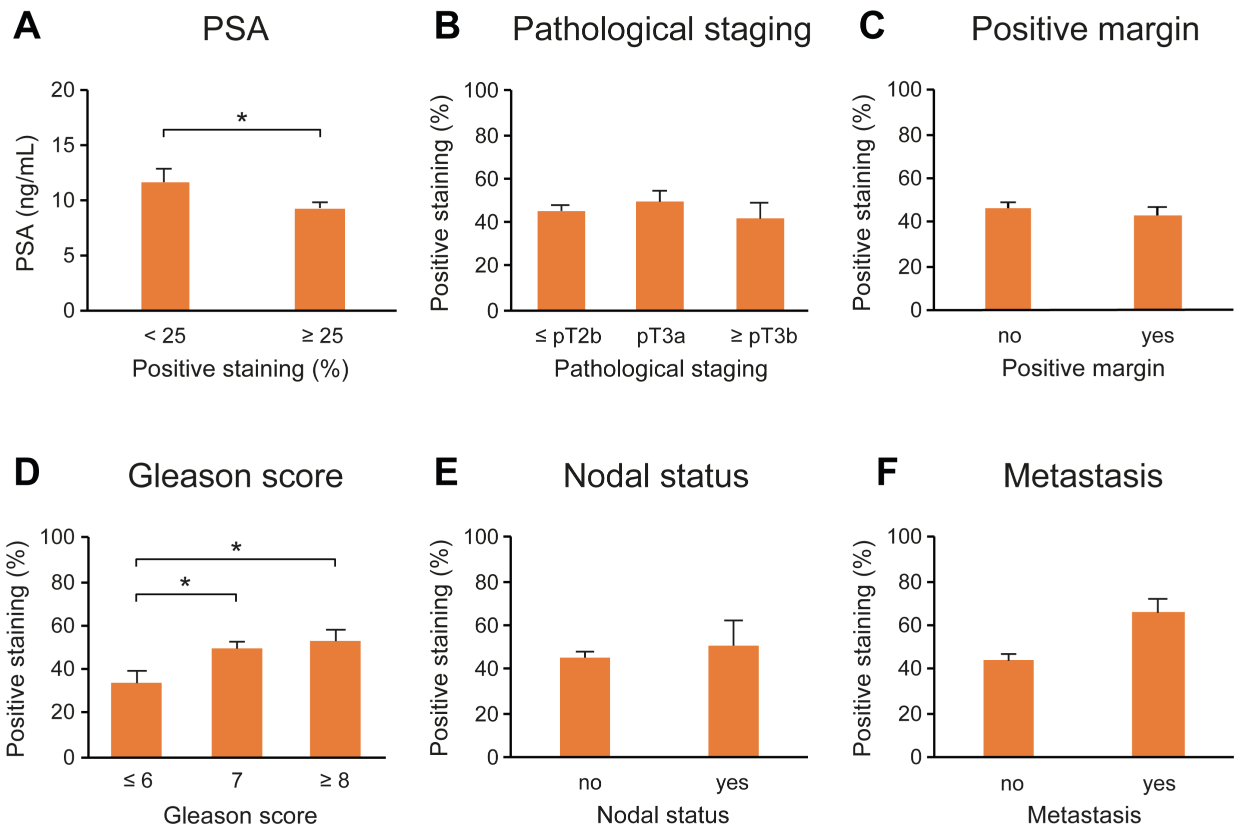

Supplementary Figure 8. UGT2B17 protein expression in primary tumors is not dependent on the functional *FOXA1* promoter polymorphism rs59678213, which influences its expression in human livers and LNCaP cells (24). Data are presented as mean ± standard error of the mean. Comparisons between groups were performed using a Kruskal-Wallis test followed by a Dunn's post-hoc test.

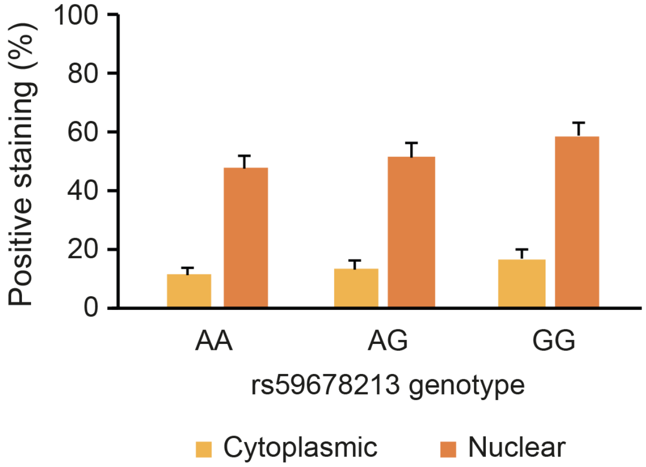

Supplementary Figure 9. Expression of *UGT2B* mRNA isoforms in PCa tissues, highlighting that several *UGT2B* transcripts are expressed at appreciable levels in 57 PCa cases from the TCGA cohort. The horizontal line represents the median, the box defines the 1st and 3rd quartiles and the whiskers extend 1.5 times the interquartile ranges. TPM: transcripts per million; UGT2B17 is indicated in red.

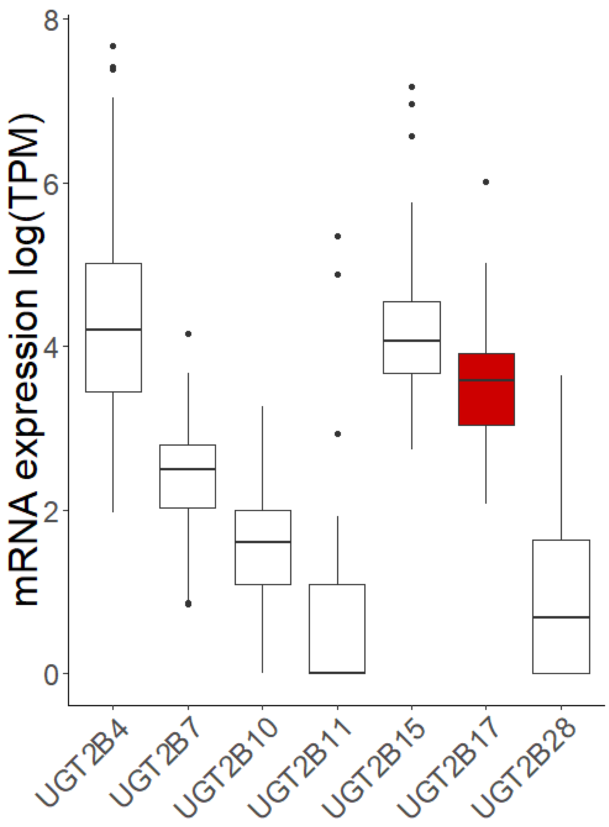

Supplementary Figure 10. Detection of *UGT2B17* alternative transcripts in LNCaP by RT-PCR. Primers (small arrows) specific to alternative exons 1b or 1c were used to amplify alternative transcript regions encompassing the junctions between exons 1b or 1c and exon 1. Direct Sanger sequencing verified each PCR product. The nucleotide sequence of the alt. exon-exon1 junction for each PCR product is provided. The blue sequence is the 3' terminal sequence of exon 1c of the *n2* transcript. Sequences in red are the 3' terminal sequences of exon 1b of n3 and n4 transcripts. The underlined sequence corresponds to the first nucleotides of the 5' untranslated region of the *UGT2B17_v1* canonical transcript (RefSeq accession: NM_001077) whereas the sequence in grey corresponds to additional sequence of exon 1 included in alt. *UGT2B17_n2-n4*.

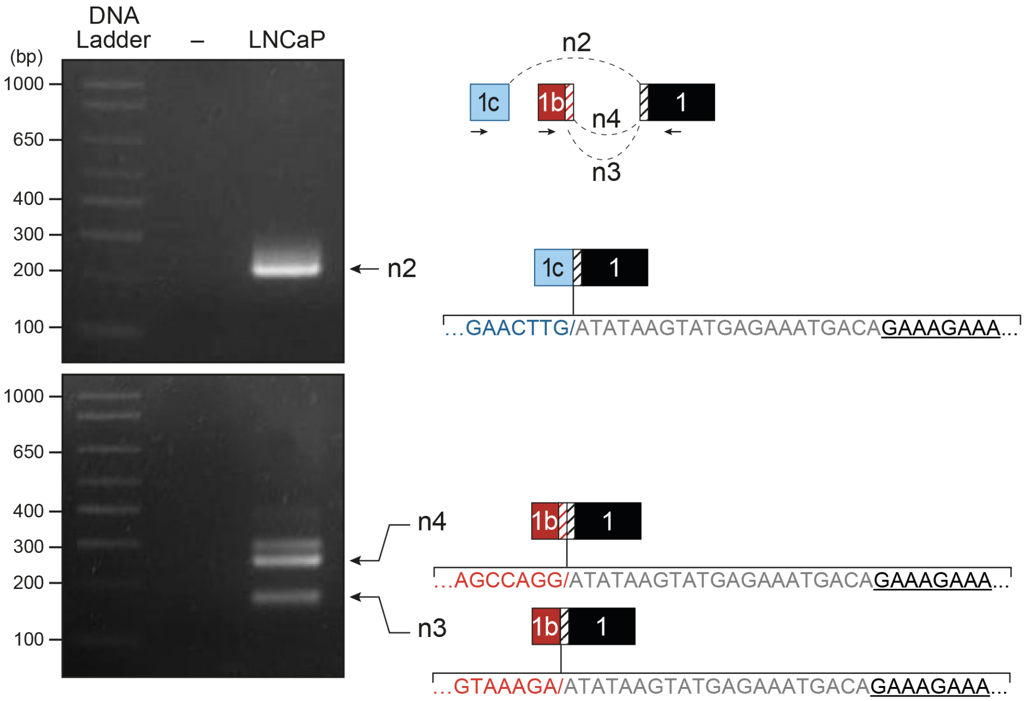

Supplementary Figure 11. Specificity of commercially available antibodies previously used to inquire UGT2B17 and UGT2B28 expression in human prostate tissues (22). Supersomes (0.5 µg) of UGT2B4, UGT2B7, UGT2B10 (1.0 µg), UGT2B15 and UGT2B17 and microsomal fractions (20 µg) of HEK293 cells expressing the recombinant human UGT2B11 and UGT2B28 were immunoblotted with **A)** the monoclonal EL-2B17mAb monoclonal antibody, B) the UGT2B17 Abcam #ab92610 polyclonal antibody and with **C)** the UGT2B28 Abcam #ab156131 polyclonal antibody. Arrows on the right of each panel identifies the UGT protein band. Microsomes from human liver (2 µg) and LNCaP cells (20 µg) were used as positive controls.

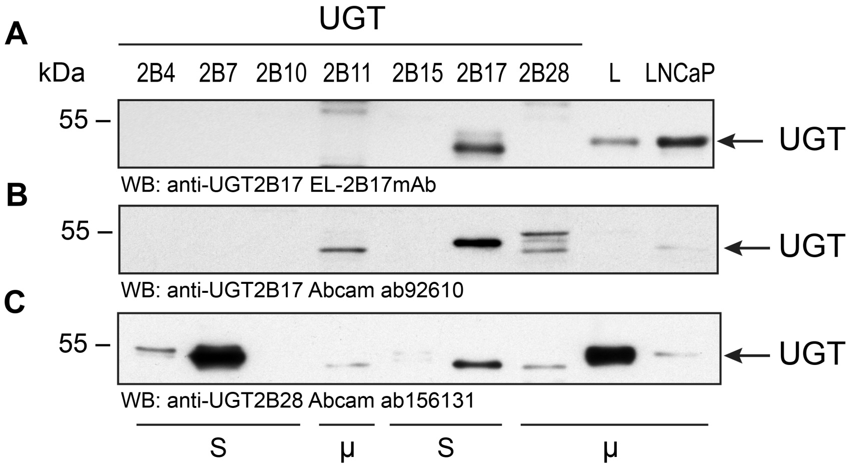

S= supersomes
µ= microsomes
L= Liver
